# Assessment of Bacteriophage Pharmacokinetic Parameters After Intra-Articular Delivery in a Rat Prosthetic Joint Infection Model

**DOI:** 10.3390/v16111800

**Published:** 2024-11-20

**Authors:** Jason Young, Mohammad Javad Shariyate, Prateek Misra, Shubham Laiwala, Ara Nazarian, Edward Kenneth Rodriguez

**Affiliations:** 1 Harvard Combined Orthopedic Residency Program, Boston, MA 02114, USA; 2 Harvard Medical School, Boston, MA 02115, USA; 3 Musculoskeletal Translational Innovation Initiative, Beth Israel Deaconess Medical Center, Boston, MA 02215, USA; 4 Department of Mechanical Engineering, Boston University, Boston, MA 02215, USA; 5 Carl J. Shapiro Department of Orthopedic Surgery, Beth Israel Deaconess Medical Center, Boston, MA 02215, USA; 6 Department of Orthopedic Surgery, Yerevan State Medical University, Yerevan 0025, Armenia

**Keywords:** bacteriophage, phage therapy, bacteriophage pharmacokinetics, prosthetic joint infections, staphylococcus epidermidis

## Abstract

Prosthetic joint infections (PJIs) are a serious complication of orthopedic surgery. Bacteriophage (phage) therapy shows promise as an adjunctive treatment but requires further study, particularly in its pharmacokinetics. Consequently, we performed a pharmacokinetic assessment of phage therapy for PJIs using a *Staphylococcus epidermidis* Kirschner wire-based prosthesis rat model. We used 52 male Sprague–Dawley rats in four groups: negative controls (no phage, sterile implant), PJI controls (bacteria, no phage), sterile phage (phages given, sterile implant), and PJI (bacteria, phages given). The PJI groups were inoculated with ~10^6^ CFU of *S. epidermidis*. The groups receiving phage were intra-articularly injected with ~10^8^ PFU of vB_SepM_Alex five days post-implantation. The rats were euthanized between 30 min and 48 h post-injection. The measured phage concentrations between the PJI rats and the sterile controls in periarticular tissues were not significantly different. In a noncompartmental pharmacokinetic analysis, the estimated phage half-lives were under 6 h (combined: 3.73 [IQR, 1.45, 10.07]). The maximum phage concentrations were reached within 2 h after administration (combined: 0.75 [0.50, 1.75]). The estimated phage mean residence time was approximately three hours (combined: 3.04 [1.44, 4.19]). Our study provides a preliminary set of pharmacokinetic parameters that can inform future phage dosing studies and animal models of phage therapy for PJIs.

## 1. Introduction

Management of orthopedic infections remains a clinical challenge. In particular, prosthetic joint infections (PJIs) are a devastating complication of arthroplasty, associated with high costs, morbidity, and decreased function [1]. One of the primary reasons for the high treatment costs and poor outcomes relates to the formation of biofilms on the implant [2]. These biofilms are resistant to antibiotic therapy and surgical debridement, leading to high recolonization and infection recurrence [1,2]. With an estimated incidence of up to 2 percent [3] and an estimated projected cost of almost USD 2 billion by 2030 [4], PJIs are associated with increased treatment costs [5,6,7], decreased patient-reported functional scores [7], and increased mortality [7,8]. Consequently, improving PJI treatment remains a research and therapeutic imperative.

Bacteriophage (phage) therapy has garnered interest as a potential adjunct for PJI eradication [9]. Phages are viruses that target bacteria [10], and many have the reported ability to penetrate and eliminate both bacterial biofilms and the bacteria residing within [11,12], thereby presenting a unique opportunity to improve the current standard of care to improve biofilm elimination and decrease infection recurrence. Further advantages include being well tolerated in vivo, high specificity to their target, and potential synergistic effects when paired with antibiotics [9]. They have been applied experimentally for various orthopedic indications, including PJIs [9,13], with preliminary studies reporting promising early results for infection eradication for *Staphylococcal* and other Gram-positive and Gram-negative infections [14,15].

Despite this interest, several challenges remain to their clinical application. Notably, little is known about optimal phage dosing regimens [16]. Unlike the vast majority of administered therapeutic agents and medications, the currently described phage administration protocols are not supported by empirical studies of phage efficacy [9,17]. The currently described regimens vary widely regarding critical administration parameters such as dosing frequency, administered titer, and treatment duration [9]. Furthermore, the literature is inconsistent in measuring efficacy, and the degree of publication bias remains unknown [17].

More fundamentally, the basic pharmacokinetic parameters of phages in vivo required to inform an evidence-based dosing protocol are poorly understood [17]. Studies of pharmacokinetic parameters are crucial to establishing an evidence base for the dosing parameters needed to build efficacious phage therapy regimens [17]. Only a single pharmacokinetic assessment of phages applied intra-articularly in a mammalian model has been reported in the literature [18]. However, this model did not assess phage behavior in an infection setting. This latter point is important as it represents the primary indication for phage therapy. Additionally, as a part of their replication cycle on target bacteria, phages have been hypothesized to self-amplify at sites of infection [19], thereby raising the possibility of increased phage persistence when applied for infections.

A foundational understanding of the pharmacokinetics of intra-articularly administered phage therapy is essential to optimize dosing and delivery parameters, thereby clarifying its therapeutic potential for PJIs. Consequently, we conducted a pharmacokinetic assessment of phage therapy for PJIs using an *S. epidermidis* rat PJI model. We chose to use a *S. epidermidis*-based PJI model as *S. epidermidis* represents one of the most common causative organisms for PJIs [20].

We hypothesized that we could generate a first set of pharmacokinetic parameters, including half-life, mean residence time, and an estimate of the terminal elimination rate constant after single-dose intra-articular phage delivery in the setting of active joint infection. Our goal was to establish pharmacokinetic parameters to enhance phage therapy research and inform clinical dosing for PJIs. We hope this information can help build an evidence base to inform future studies of phage dosing and efficacy.

## 2. Materials and Methods

### 2.1. Bacterial Strain

We employed *S. epidermidis* ATCC 35984 (ATCC, Manassas, VA, USA), a known biofilm-forming, ampicillin-sensitive strain [21] derived from a clinical isolate [22]. Bacterial colonies were cultured for all the experiments using brain heart infusion (BHI) broth (AG Scientific Incorporated, San Diego, CA, USA). The bacterial samples were stored in a BHI broth and 16.7% (*v*/*v*) of glycerol at −80 °C.

### 2.2. Phage Preparation and Handling

We have previously demonstrated the in vitro susceptibility and dose-dependent response of our bacterial strain to an obligate lytic phage, vB_SepM_Alex, a myovirus obtained from the Leibniz Institute DSMZ (Braunschweig-Süd, Germany) known to form clear lytic plaques on our bacterial strain [23]. Phages were propagated in liquid BHI cultures of ATCC 35984, as previously described [23,24]. The lysate purification method used is detailed in a previous study from this research group [23]. The phage preparations were sterilely isolated from the bacterial lysate and then diluted to the appropriate concentration for phosphate-buffered saline (PBS) administration. The phage concentrations were enumerated using a double agar overlay method [25] and expressed in plaque-forming units per milliliter (PFU/mL). The phages were subsequently stored at 4 °C.

### 2.3. Phage Dosing

In the absence of established optimal dosing guidelines, the ideal phage titer, dosing volume, and dosing frequency needed to elicit a therapeutic response are unknown [9]. We prepared a high-titer phage (10^9^ PFU/mL) for subsequent delivery to maximize phage detection and potential observed treatment response. We previously observed the susceptibility of this bacterial strain to the phage employed in this study, as well as a dose dependency in vitro [23].

However, given our objective of building the first set of pharmacokinetic parameters for intra-articular phage dosing in the infection setting, a single dose of phage was administered (see “Rat Treatment Groups and Surgical Procedures” below) in our experiments. While used clinically to maximize therapeutic effect [9], repeated dosing was deferred in this study to simplify the experimental protocol and facilitate the identification of pharmacokinetic parameters. As such, it was anticipated that a measurable reduction in bacterial bioburden might not occur between our experimental groups.

### 2.4. Bacterial Preparation

Overnight cultures of transformed bacteria were diluted 1:50 in fresh BHI media and incubated until the early exponential phase (OD_600_ ~ 0.3). The bacteria were pelleted at 3400 g and washed with PBS three times before resuspension in 100 μL of PBS. The bacteria were diluted in PBS before delivery to administer approximately 10^6^ CFU per injection (see below). The bacterial concentrations were estimated using spectrophotometric optical density measurements (OD_600_ ~ 0.2) and confirmed via plate culture in triplicate.

### 2.5. Rat Treatment Groups

All the procedures were performed in 12- to 13-week-old male Sprague–Dawley rats (Charles River Laboratories, Wilmington, MA, USA).

In total, 52 rats underwent placement of a prosthetic model in the right distal femur. The animals were divided into four groups: negative controls (no phage delivery, sterile implant, n = 4), PJI controls (no phage delivery, PJI model, n = 6), sterile phage (phages given, sterile implant, n = 21), and PJI (phage given, PJI model, n = 21).

For the groups receiving the phage, sample sizes were determined based on a power of 0.8 to detect a 2-fold difference in phage concentration between groups (3 animals per time point per group), which aligns with prior reports [18].

### 2.6. Rat Surgical Procedures

**Figure 1:**
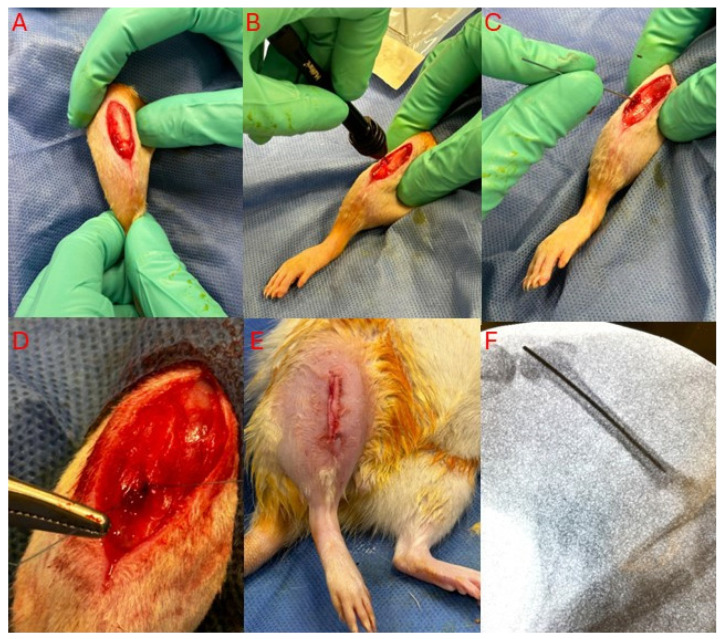
Prosthesis Insertion Technique. (**A**) A 3 cm skin incision is made overlying the knee. (**B**) Subsequently, a hand drill is used to open the distal femoral cortex through a transtendinous poke hole. (**C**) A 0.8 mm K-wire is then inserted through the drill hole into the intramedullary canal. The wire is advanced past the isthmus for adequate purchase. (**D**) The poke hole is closed with a single 4-0 nylon stitch. (**E**) The skin is closed with a 4-0 nylon suture. (**F**) A postoperative fluoroscopic image demonstrating intramedullary placement of the implant with intra-articular exposure of the distal end.

For the prosthetic implantation, a modified version of a previously described distal femoral implant insertion technique was employed [26], whereby implantation was performed transtendinously through the patellar tendon to minimize capsular insult (Figure 1). A full description of the surgical technique is provided in Supplemental File S1. The ARRIVE checklist is provided in Supplemental File S2, with additional ARRIVE checklist details provided in Supplemental File S3.

For the infected groups, 25 μL of ~10^6^ CFU of transformed *S. epidermidis* in PBS was injected into the joint with a 30-gauge needle after capsular closure but before skin closure.

At five days postoperatively, the animals were anesthetized, and 100 μL of ~10^9^ PFU/mL of phage was percutaneously injected into the intra-articular space of the right knee via a 30-gauge needle for the groups receiving the phage. The chosen injection volume was near the maximal limit of reported knee injection volumes in the literature [27] and, along with the high phage titer administered, was selected to maximize the phage delivery and facilitate detection. PBS was administered to the control groups. Intra-articular needle placement was confirmed fluoroscopically to ensure intra-articular delivery. Subsequently, in the groups receiving the phage, three rats were euthanized via CO_2_ euthanasia at each of the following time points post-phage injection: 30 min, 1 h, 2 h, 4 h, 8 h, 24 h, and 48 h. For the sterile control group, a single rat was sacrificed on each of the postoperative days 5, 6, and 7, without phage administration. For the PJI controls, three animals each were euthanized on postoperative days 5 and 6, respectively, without phage administration.

### 2.7. Tissue Harvest and Processing

Immediately following euthanasia, blood samples were collected via cardiac puncture, as described previously [28]. The blood was stored in BD Vacutainer blood collection tubes containing sodium citrate (Becton Dickinson, Franklin Lakes, NJ, USA) before processing. Subsequently, the periarticular tissues of the operative limb were sterilely dissected, and the overlying muscle was removed, leaving the joint capsule intact. The tibia and femur were cut transversely, just distal and proximal to the margins of the joint capsule, respectively, with the inserted K-wire cut at the same level.

The cut K-wire was weighed, combined with ten times its weight in PBS, and subsequently vortexed and sonicated in a water bath (FS20D Ultrasonic Cleaner, Fisher Scientific, Hampton, NH, USA) according to a previously defined protocol for murine model prosthesis sonication [29].

The periarticular tissues were combined with weight-adjusted quantities of PBS and were subsequently sterilely morselized and then homogenized in a modified version of the previously described techniques [30], using a Fisher Scientific 850 Homogenizer (Fisher Scientific, Hampton, NH, USA) set at 5000 RPM for ten one-minute cycles.

### 2.8. Phage Enumeration

Our primary outcome was phage concentration (PFU/mL) after phage administration. Samples of blood and homogenized tissues were centrifuged at 16,000× *g* for one minute, and the supernatants were subsequently passed through 0.22-micron filters. The blood and tissue homogenate supernatants, as well as sonicate, were subsequently serially diluted in SM Buffer (Thermo Fisher Scientific, Waltham, MA, USA), and the phage concentrations (PFU/mL) were enumerated after overnight incubation at 37 °C on ATCC 35984 lawns grown via double agar overlay [25]. We defined our limit of detection as the phage concentration for which a single plaque could be identified from blood (2 log_10_ PFU/mL), sonicate (3 log_10_ PFU/mL), or processed harvested tissues (2.70 log_10_ PFU/mL) from the plated culture. All the phage plating was performed in triplicate.

### 2.9. Bacterial Enumeration

Blood, homogenized tissues, and sonicate were serially diluted in PBS and plated on BHI agar plates. Bacterial concentrations (CFU/mL) were enumerated after overnight incubation at 37 °C. We defined our limit of detection as the bacterial concentration for which a single colony could be identified from blood (2 log_10_ CFU/mL), sonicate (3 log_10_ CFU/mL), or processed harvested tissues (2.70 log_10_ CFU/mL) from the plated culture. The bacterial plating was performed in triplicate.

### 2.10. Post-Harvest Bacterial Assessment

To help confirm *S. epidermidis* infection in our rat model, periarticular tissues from all the rats receiving bacterial inoculations were plated, and subsequently, isolated bacterial colonies were subjected to Gram stain, coagulase, and catalase testing using standard techniques [31,32,33].

### 2.11. Statistics

Descriptive statistics and Wilcoxon Rank Sum Tests were calculated in R (RStudio, version 2024.04.01+748, Posit Software, PBC, Boston, MA, USA). The figures were generated in GraphPad (GraphPad Software Inc., Dotmatics, Boston, MA, USA). Pharmacokinetic modeling was performed using the PKSolver [34] plug-in for Microsoft Excel (Microsoft, Redmond, WA, USA). The level of significance was set at 0.05.

### 2.12. Ethics and Institutional Review

All the animal procedures herein described were conducted in accordance with the Institutional Animal Care and Use Committee at our institution (Protocol #038-2023, approved 16 January 2024).

## 3. Results

### 3.1. Post-Implantation, Euthanasia, and Harvest

After initial implantation, a single rat from the sterile control group died immediately postoperatively. No intra-operative surgical concerns or complications were noted. Postmortem assessment suggested the cause was likely attributable to hypersensitivity to the administered anesthetic. All the other rats survived until the tissue harvest (51/52). Subsequent analyses were performed on the remaining 51 rats. The clinical characteristics immediately before euthanasia are provided in Supplemental File S4. The mean weight of harvested periarticular tissue was 1.41 ± 0.21 g, and the mean weight of harvested implants was 0.07 ± 0.02 g.

### 3.2. Bacterial and Phage Enumeration

All the animals were dissected immediately upon euthanasia at the aforementioned time points. The animals were euthanized and dissected individually to avoid tissue processing delays.

**Figure 2:**
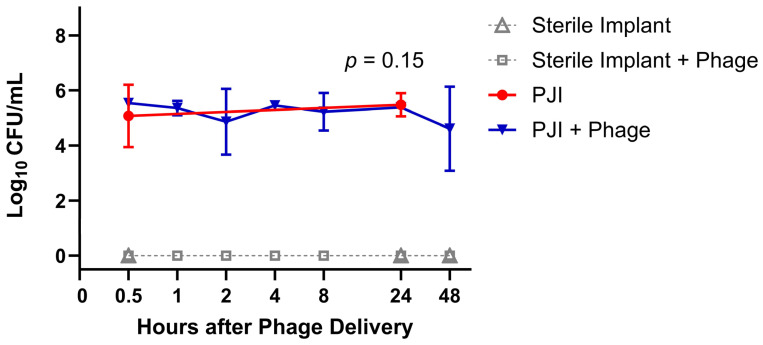
Bacterial Concentrations in Periarticular Tissue Over Time. Displaying the mean count and range. *p*-value from the Wilcoxon Rank Sum Test. Phage delivery was performed 5 days after implant insertion and bacterial inoculation.

All the PJI rats demonstrated infection establishment (27/27) based on the plated culture of periarticular tissues. The mean CFUs in the PJI rats receiving phage therapy ranged from 5.2 to 5.5 log_10_ CFU/mL (Figure 2). Bacterial concentrations were not significantly different at 0.5 and 24 h between the PJI groups receiving phage versus saline control (Wilcoxon Rank Sum Test, Z = −1.43, *p* = 0.15). No rats in the Sterile Implant Groups (25/25) grew bacteria from periarticular tissues on the plated culture.

**Figure 3:**
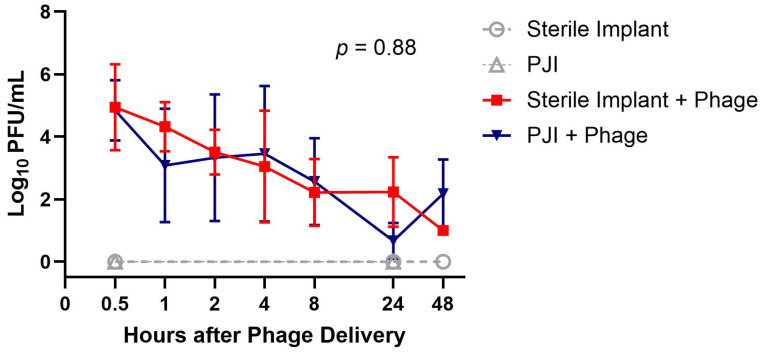
Phage Concentrations in Periarticular Tissue Over Time. Displaying the mean count and range. *p*-value from the Wilcoxon Rank Sum Test.

Maximum mean phage concentrations were recorded at the first time point of 0.5 h (5.3 log_10_ PFU/mL in the PJI group and 5.4 log_10_ PFU/mL in the sterile controls) (Figure 3). The measured phage concentrations between the sterile and PJI groups were not significantly different over time (Wilcoxon Rank Sum Test, Z = −0.15, *p* = 0.88).

No bacteria could be isolated from blood for any group. The mean phage counts isolated from blood and mean phage and bacteria counts isolated from sonicated fluid were below the detection limit of our assays at nearly all time points and were not further analyzed.

### 3.3. Noncompartmental Pharmacokinetic Analysis

We performed a noncompartmental pharmacokinetic analysis of the delivered phage in periarticular tissues in the Sterile Implant Group (n = 21), PJI Group (n = 21), and a combined analysis (n = 42). The estimated half-lives were <6 h for all rats (combined: 3.73 [IQR, 1.45, 10.07], Sterile Group: 5.59 [3.45, 14.57], PJI Group: 1.87 [1.01, 6.72]). The maximum phage concentrations were reached within 2 h after administration (combined: 0.75 [0.50, 1.75], Sterile Group: 1.00 [0.75, 1.50], PJI Group: 0.50 [0.50, 1.25]). The estimated mean residence time (MRT) of the phages was approximately 3 h (combined: 3.04 [1.44, 4.19], Sterile Group: 3.06 [1.99, 17.09], PJI Group: 3.02 [1.78, 3.79]). Complete pharmacokinetic parameters are provided in Table 1.

### 3.4. Post-Harvest Bacterial Assessment

The Gram stain, catalase, and coagulase testing on bacteria isolated from the periarticular tissues demonstrated all the samples grew Gram-positive, catalase-positive, and coagulase-negative organisms (Supplemental File S5). These findings suggest the growth of coagulase-negative Staphylococcus consistent with our initial inoculating organism and suggest against predominant colonization with contaminant species.

## 4. Discussion

Despite a growing interest in the therapeutic use of phages, their application has been hindered by a limited understanding of phage pharmacokinetics [17,35,36,37,38]. While several prior phage pharmacokinetic assessments in murine models have been described for pulmonary [39], intraperitoneal [40], or bacteremic infection [41], a dedicated pharmacokinetic assessment of phage application for PJIs has yet to be described. A single pharmacokinetic assessment of intra-articularly administered phages has been performed in a rabbit model [18], though no bacterial infection was established in this model. Consequently, we used a rat PJI model to perform one of the first characterizations of basic pharmacokinetic parameters for intra-articularly applied phages in active joint infection.

Our findings suggest half-lives of approximately 2 to 6 h after intra-articular phage administration. This range is consistent with previously reported pharmacokinetic assessment of phage half-lives in other settings [18], where phage half-lives were estimated to range from 2 to 5 h for murine models across different species and systemic delivery methods [37,38,41]. These relatively short half-lives are hypothesized to be attributable to the rapid clearance of phage via the immune system via circulating factors in the blood [38] or monocyte-mediated pathways, such as clearance by splenic and liver macrophages [42,43]. However, it remains unclear whether such mechanisms are the principal mediators of phage clearance after intra-articular administration. Indeed, few studies to date have examined phage clearance in the intra-articular environment. Some have hypothesized, for instance, that chondroitin sulfate, found in abundance in articular cartilage [43], may play a role in the joint, while Toll-like receptors [44] and antibody or T cell responses [43,44] may all participate in phage clearance outside of systemic circulation. Additionally, how phage therapy–immune system interactions impact their clearance is poorly understood. The existing literature suggests divergent effects of phages on immune system function; for instance, phages have been described to impair macrophage and dendritic cell phagocytosis and reduce mammalian cytokine production, while other reports suggest synergistic effects with neutrophils on bacterial clearance and upregulation of T cell activity [43]. Given the conflicting reports and limited data, future studies are needed to clarify the immune system’s role in modulating phage persistence and to determine the specific mechanisms responsible for intra-articular phage clearance. However, regardless of the mechanism, our findings and the previous literature empirically support a rapid mammalian clearance of administered phage, limiting its in vivo persistence. As such, the pharmacokinetic parameters outlined in this study would support the recent recommendations for repeated phage dosing every few hours [17,45,46] or dosing through sustained release mechanisms, such as through phage-containing hydrogels [9,47,48,49], to mitigate the effects of rapid phage clearance and help ensure measurable therapeutic effects when employing phage therapy.

In our study, the phages administered in the PJI Group had a shorter half-life (1.87 h) than those in the Sterile Implant Group (5.59 h). While our study design and analysis were not powered to enable a statistical comparison between the half-lives of these two groups, our findings suggest some phages may exhibit shorter half-lives in the presence of host bacteria. This observed half-life reduction in the presence of infection has previously been documented. Dhungana et al. [40] noted in their murine model of intraperitoneal and oral administration of phage against drug-resistant *Klebsiella* that phage half-life was measured to be 8 h after oral delivery in a sterile model but only 5 h when administered in an infection model, possibly due to more rapid clearance by a stimulated immune system [40]. However, these findings contrast with other studies that have documented prolonged phage half-lives in the presence of infection [39], possibly due to replication of phages in situ. In addition to the conflicting reports on phage-immune interactions outlined above, these discrepant reports highlight the complexity of understanding phage biology in vivo and the need for corroborative studies to clarify phage behavior and interplay with host immune responses in the infection setting.

Interestingly, the mean phage counts from blood were below the limit of detection. This may be partly due to low systemic penetration of phages after local delivery, which has previously been described after intra-articular delivery [18]. The reasons for this remain unclear but may be attributable to the contained nature of the joint and limited movement of phage through capsular tissue. Alternatively, the rapid clearance of phage from systemic circulation through pre-existing anti-phage antibodies [50] or clearance by host immune cells [43] may also contribute to low circulating phage counts. It remains unclear whether intra-articular delivery elicits a different immune response compared to systemic phage delivery and, if so, how such a difference might impact pharmacokinetic parameters. As our study was focused on understanding the pharmacokinetics of intra-articularly delivered phage, we did not separately investigate for the presence of phage-neutralizing antibodies or the development of antibodies specific to our administered phage, nor did we compare immune responses between systemic and intra-articularly delivered phage. Future work will be needed to focus on in vivo immune responses after phage administration across different delivery strategies.

Of note, we limited our pharmacokinetic assessment to a single dose of administered phage in a small-animal model of PJI. We recognize that this single phage dose was not optimized for bacterial reduction, as we observed no difference in bacterial counts in harvested tissues between the groups receiving phage and bacterial controls. We have previously demonstrated the in vitro efficacy of vB_SepM_Alex on our tested strain of *S. epidermidis* and found bacterial reduction to depend on the titer of administered phage [23]. The prior literature also suggests repeated or prolonged phage exposure is needed to observe therapeutic effects in an in vivo setting [17]. As such, while a single dose was likely insufficient to yield a significant reduction in bacterial burden, it facilitated the measurement of phage pharmacokinetic parameters (half-life, area under the concentration curve, mean residence time, etc.), which was the principal goal of this analysis.

### Limitations

Our study was not without limitations. First, while this murine model is widely used [26,51,52], it does not replicate important aspects of prosthesis design, including bone-on-cement and metal-on-polyethylene interfaces. Second, due to the small size of our model, implant sonicates could not yield detectable phage or bacteria, and repeated sampling within animals was not possible. Consequently, variability within organisms may introduce bias and increase the variability of observed phage or bacterial counts. Third, despite multiple attempts at plasmid transformation, we could not use a marker to confirm infection with our *S. epidermidis* strain. *S. epidermidis* transformation is not achievable for many strains [53]. As such, while our post hoc assessments suggest *S. epidermidis* infection, we cannot rule out infection with a contaminant coagulase-negative *Staphylococcal* strain. Future studies should consider using *S. aureus*-based bacterial PJI models that are amenable to plasmid transformation or PCR-based methods to confirm infection establishment with the target bacterial strain. Fourth, we chose to assess a single phage–bacterial pair. While our findings are consistent with prior studies, future work across bacterial and phage species is needed to build a robust understanding of phage pharmacokinetics in vivo. Additionally, future studies should evaluate how other parameters affect phage pharmacokinetics, including phage size [17,42], morphology [36], immune response [35], and phage replicative ability [17]. Fifth, endotoxin levels were not measured in the phage lysates before the phage administration. Endotoxin testing of phage lysates will be needed prior to any future studies involving phage administration for humans. Finally, for bacterial and phage enumeration, we employed plate-based assays. While plate-based plaquing assays are the mainstay for detecting phage particles [54], this approach may not be an adequately sensitive detection modality. While qPCR may be an alternative, prior studies have reported discrepancies between qPCR and plaque assay-based phage enumeration methods [38], as the former cannot differentiate phage fragments from functioning phage [35,38]. Consequently, future studies should explore how different phage administration techniques impact immune response and phage clearance and consider alternative phage detection techniques [54] to produce more robust and sensitive phage detection protocols.

## 5. Conclusions

Our study represents one of the first to assess the pharmacokinetics of phages for intra-articularly applied phages in the setting of prosthetic joint infection. We identified basic pharmacokinetic parameters to inform future in vivo dosing studies and clinical applications in this disease context. Our findings suggest relatively short phage persistence in vivo, supporting emerging evidence that repeated or continuous phage dosing may be more efficacious for bacterial reduction. Future studies should consider replicating pharmacokinetic assessments across different bacteria–phage pairs and include larger animal models to enable repeated intra-articular sampling within individual animals over time. Ultimately, we hope this line of work can help inform clinical applications of phage therapy as an emerging adjunct for PJI management.

## Figures and Tables

**Table 1:** Pharmacokinetic Parameters of Phage vB_SepM_Alex Following Intra-Articular Injection (~108 PFU).

Parameter	Unit	Combined Pharmacokinetic Assessment (Median [IQR]) (n = 42)	Sterile Implant + Phage (Median [IQR]) (n = 21)	PJI + Phage (Median [IQR]) (n = 21)
λz	1/h	0.25 [0.08, 0.49]	0.12 [0.08, 0.33]	0.37 [0.22, 2.63]
t_1/2_	h	3.73 [1.45, 10.07]	5.59 [3.45, 14.57]	1.87 [1.01, 6.72]
t_max_	h	0.75 [0.50, 1.75]	1.00 [0.75, 1.50]	0.50 [0.50, 1.25]
C_max_	PFU/mL	1.61 × 10^5^ [2.75 × 10^4^, 2.83 × 10^5^]	2.05 × 10^4^ [1.48 × 10^4^, 3.94 × 10^5^]	2.73 × 10^5^ [1.61 × 10^5^, 2.80 × 10^5^]
AUC_0–t_	PFU/mL*h	1.27 × 10^5^ [7.79 × 10^4^, 3.77 × 10^5^]	6.95 × 10^4^ [5.09 × 10^4^, 2.61 × 10^5^]	1.50 × 10^5^ [1.27 × 10^5^, 7.69 × 10^5^]
AUC_0–∞_	PFU/mL*h	1.31 × 10^5^ [8.06 × 10^4^, 3.78 × 10^5^]	7.01 × 10^4^ [6.54 × 10^4^, 2.62 × 10^5^]	1.50 × 10^5^ [1.31 × 10^5^, 7.75 × 10^5^]
AUMC_0–∞_	PFU/mL*h^2^	3.75 × 10^5^ [2.46 × 10^5^, 1.52 × 10^6^]	4.12 × 10^5^ [3.13 × 10^5^, 1.15 × 10^6^]	3.39 × 10^5^ [2.10 × 10^5^, 3.36 × 10^6^]
MRT_0–∞_	h	3.04 [1.44, 4.19]	3.06 [1.99, 17.09]	3.02 [1.78, 3.79]
Vz/F_obs_	(PFU)/(PFU/mL)	2.09 × 10^3^ [1.34 × 10^3^, 2.61 × 10^3^]	2.68 × 10^3^ [2.23 × 10^3^, 2.93 × 10^4^]	1.19 × 10^3^ [6.64 × 10^2^, 1.80 × 10^3^]
Cl/F_obs_	(PFU)/(PFU/mL)/h	7.79 × 10^2^ [3.32 × 10^2^, 1.29 × 10^3^]	1.43 × 10^3^ [8.23 × 10^2^, 1.54 × 10^3^]	6.66 x10^2^ [3.68 × 10^2^, 7.79 × 10^2^]

λz: terminal elimination rate constant estimate. t_1/2_: half-life estimate. t_max_: time of maximum plasma concentration. C_max_: maximum plasma concentration estimate. AUC_0–t_: Area under the concentration-time curve (total exposure estimate) from zero to the last measured time point (48 h). AUC_0–∞_: area under concentration-time curve (total exposure estimate) over time. AUMC_0–∞_: area under the moment curve, from zero to infinity. MRT_0–∞_: estimate of mean residence time. Vz/F_obs_: apparent volume of distribution. Cl/F_obs_: apparent total plasma clearance.

## Data Availability

The raw data supporting the conclusions of this article will be made available by the authors upon request.

## Supplementary Materials

The following supporting information can be downloaded at: https://www.mdpi.com/article/10.3390/v16111800/s1, File S1: Animal Care and Surgical Procedures; File S2: ARRIVE Checklist; File S3: Additional ARRIVE Checklist Details; File S4: Clinical Characteristics of Study Rats 5 Days Postoperatively; File S5: Assessment of Bacteria Isolated from Periarticular Tissues.
