# Supplementary material for: Assessment of Bacteriophage Pharmacokinetic Parameters After Intra-Articular Delivery in a Rat Prosthetic Joint Infection Model
Source: Viruses. 2024 Nov 20;16(11):1800. doi: 10.3390/v16111800 (PMC11598970; doi:10.3390/v16111800)
Supplement: Supplementary file 1 [file viruses-16-01800-s001.zip › S4 Clinical Characteristics of Study Rats 5 Days Postoperatively.docx]

**Supplemental File 4:** Clinical Characteristics of Study Rats 5 Days Postoperatively

|  | Sterile Implant Group (n=4)* (%) | Sterile Implant + Phage Group (n=21) (%) | PJI Group (n=6) (%) | PJI + Phage Group (n=21) (%) |
| --- | --- | --- | --- | --- |
| Premature Mortality | 1 (25%) | 0 (0%) | 0 (0%) | 0 (0%) |
| Toxic Appearance on Clinical Examination | 0 (0%) | 0 (0%) | 0 (0%) | 0 (0%) |
| Bearing Weight on Operative Limb | 3 (100%) | 21 (100%) | 6 (100%) | 21 (100%) |
| Wound Closed | 3 (100%) | 21 (100%) | 6 (100%) | 21 (100%) |
| Presence of Peri-Incisional Erythema | 0 (0%) | 0 (0%) | 0 (0%) | 3 |
| Presence of Frank Purulence | 0 (0%) | 0 (0%) | 0 (0%) | 0 (0%) |

*One animal died immediately postoperatively. Clinical assessment performed for remaining 3 rats.
