# Supplementary material for: Assessment of Bacteriophage Pharmacokinetic Parameters After Intra-Articular Delivery in a Rat Prosthetic Joint Infection Model
Source: Viruses. 2024 Nov 20;16(11):1800. doi: 10.3390/v16111800 (PMC11598970; doi:10.3390/v16111800)
Supplement: Supplementary file 1 [file viruses-16-01800-s001.zip › S5 Assessment of Bacteria Isolated from Periarticular Tissues.docx]

**Supplemental File 5:** Assessment of Bacteria Isolated from Periarticular Tissues

|  | Number of Samples (%) |
| --- | --- |
| Positive Gram Stain | 27 (100%) |
| Positive Catalase Test | 27 (100%) |
| Positive Coagulase Test | 0 (0%) |
