# Supplementary material for: Assessment of Bacteriophage Pharmacokinetic Parameters After Intra-Articular Delivery in a Rat Prosthetic Joint Infection Model
Source: Viruses. 2024 Nov 20;16(11):1800. doi: 10.3390/v16111800 (PMC11598970; doi:10.3390/v16111800)
Supplement: Supplementary file 1 [file viruses-16-01800-s001.zip › S1 Animal Care and Surgical Procedures.docx]

***Animal Care and Surgical Procedures:***

Before starting animal procedures, a protocol outlining the research described in this study was prepared and registered with our institution’s Institutional Animal Care and Use Committee. The protocol was approved before the initiation of animal experimentation. (Protocol# 038-2023).

All procedures were performed in male Sprague-Dawley rats (Charles River Laboratories, Wilmington, MA), aged 12-13 weeks. All animal-based procedures and experiments were conducted in accordance with the Institutional Animal Care and Use Committee.

All rats underwent a minimum of 48 hours of acclimation before procedures. Free access to food and clean water was provided. Environmental enrichment in the form of nesting material and rat tunnels (Bio-Serv, Flemington, NJ) was provided to all rats. Rats were housed singly after prosthesis insertion.

For the prosthetic insertion, all animals received isoflurane inhaled anesthesia as well as 1.2 mg/kg of extended-release buprenorphine before the procedure started. Adequate anesthesia was confirmed via a pinch test prior to incision. After induction of anesthesia, the right hindlimb was shaved and cleaned with betadine and 70% ethanol. A surgical timeout was performed prior to the incision. All procedures were performed using a sterile technique.

After administering sustained-release buprenorphine, a 3 cm skin incision was made over the lateral aspect of the right hindlimb. Subsequently, a 1 mm drill bit was manually advanced directly through the patellar tendon just inferior to the inferior pole of the patella and through the cortical bone in the intercondylar notch via tactile feedback. Subsequently, a 0.8 mm stainless steel Kirschner wire (K-wire) (GreatLH Orthopedics, Chengdu, China) was advanced through the cortical defect. The wire was subsequently advanced until it engaged the cortical bone of the proximal femur. Placement was confirmed via fluoroscopic imaging intraoperatively. The wire was backed out by 5 mm, cut, then readvanced. Care was taken to ensure at least 1 mm of wire was left prominent within the intra-articular space. The defect in the tendon was closed with a single 4-0 nylon suture.

Subsequently, prior to skin closure, PBS or bacterial inoculum was administered with a 30-gauge needle after capsular closure but prior to skin closure. The skin was subsequently closed with 4-0 nylon suture.

Sterile dressings were applied, and animals were monitored postoperatively in their cages and placed on heated pads for recovery. Once awake, the animals were returned to the animal facility. Animals were subsequently evaluated daily per animal safety protocols for signs suggestive of care escalation or consideration of human endpoints (none).

- Human endpoints included:
- Decreased water and/or food consumption
- Up to 20% weight loss
- Abnormal posture
- Reluctance to move
- Dehydration
- Body Score 2-/5 (skin turgor)
- Failure to groom/Roughened hair coat
- Decreased activity or responsiveness
- Other: Systemic Infection
