# Supplementary material for: Assessment of Bacteriophage Pharmacokinetic Parameters After Intra-Articular Delivery in a Rat Prosthetic Joint Infection Model
Source: Viruses. 2024 Nov 20;16(11):1800. doi: 10.3390/v16111800 (PMC11598970; doi:10.3390/v16111800)
Supplement: Supplementary file 1 [file viruses-16-01800-s001.zip › S3 Additional ARRIVE Checklist Details.docx]

**ARRIVE Checklist Additional Details**

**Sample Size:**

- 2b: Explain how the sample size was decided. Provide details of any a priori sample size calculation, if done.

The goal of the study was to perform an initial pharmacokinetic assessment of bacteriophages administered intraarticularly in the setting of PJI. As such, a sample size estimation based on a power calculation was not performed, as no comparative assessment was planned for our study. Instead, sample sizes were determined based on previous literature, specifically a single prior intraarticular pharmacokinetic assessment performed in rabbits, in which the authors used three rabbits (n=3) per time point assessed.^13^

**Inclusion and Exclusion Criteria:**

- 3a: Describe any criteria used for including and excluding animals (or experimental units) during the experiment, and data points during the analysis. Specify if these criteria were established a priori. If no criteria were set, state this explicitly.

*A priori*, all healthy male Sprague-Dawley rats aged 12-13 weeks were eligible for inclusion in our study. Exclusion criteria included animals with obvious signs of active or prior infection (n=0) or those with evidence of prior limb amputation (n=0). No additional inclusion or exclusion criteria were set.

**Randomization:**

- 4a: State whether randomisation was used to allocate experimental units to control and treatment groups. If done, provide the method used to generate the randomisation sequence.

Formal randomization was not used to allocate animals to different experimental groups, as animals were ordered in stages and each experimental group was ordered as a batch of animals together. However, animals were assigned at random by study staff to individual timepoints.

- 4b: Describe the strategy used to minimise potential confounders such as the order of treatments and measurements, or animal/cage location. If confounders were not controlled, state this explicitly.

To minimize confounders, all animals were ordered from the same vendor and housed in the same facility, and given the same environmental enrichment, food, and water. All prosthesis insertions were performed in the morning and pilot tests were conducted to optimize insertion technique and protocols prior to starting on experimental animals. The same equipment as used for all procedures, and implants were manufactured from a single company. The same batches of phage and bacteria were used on all animals undergoing surgery on a given day. All procedures for different replicates were performed on different days using different phage and bacterial batches. To avoid contamination, sterile controls and the sterile group underwent prosthesis insertion and tissue harvest prior to PJI group prosthesis insertion.

**Blinding:**

- 5: Describe who was aware of the group allocation at the different stages of the experiment (during the allocation, the conduct of the experiment, the outcome assessment, and the data analysis).

The study team (JY, JS, SL, PM) were aware of group allocations during the allocation and conduct of the experiment. Group allocations during outcome assessment and data analysis were known to part of the study team (JY and SL).

**Experimental Animals:**

- 8b: Provide further relevant information on the provenance of animals, health/immune status, genetic modification status, genotype, and any previous procedures.

All animals were obtained from Charles River Laboratories (Charles River Laboratories International, Inc, Wilmington, MA). We used a strain of healthy, non-immunocompromised outbred Sprague-Dawley Rats (CD-001). No prior procedures were performed on these animals.

**Background**

- 12b: Explain how the animal species and model used address the scientific objectives and, where appropriate, the relevance to human biology.

We used Sprague-Dawley rats due to their relative docility, ease of handling, and institutional familiarity with this strain. We used rats in this study as they represent the smallest commonly used laboratory animal that enables us to employ a model for prosthetic joint infection while permitting reliable intra-articular injection. Mouse models are too small for us to reliably implant a prosthesis and perform intraarticular injections. By recapitulating intraarticular infection, use of a rat model enables us to provide a first assessment of *in vivo* phage pharmacokinetics for a PJI model that can help inform dosing and pharmacokinetic studies in larger animal models and human phage therapy applications.

**Data Access:**

- 20: Provide a statement describing if and where study data are available.

Data from this study will be made available upon reasonable request by the corresponding author.
